# Penetration of Carbon Nanotubes into the Retinoblastoma Tumor after Intravitreal Injection in LH${}_{\mathrm{BETA}}$T${}_{\mathrm{AG}}$ Transgenic Mice Reti-noblastoma Model

**DOI:** 10.18502/jovr.v15i4.7778

**Published:** 2020-10-25

**Authors:** Hakan Demirci, Yichun Wang, Qiaochu Li, Cheng-mao Lin, Nicholas A Kotov, Anna Beatriz Diniz Grisolia, Jay L. Guo

**Affiliations:** ^1^Department of Ophthalmology and Visual Sciences, Kellogg Eye Center, University of Michigan Ann Arbor, MI, USA; ^2^Biointerfaces Institute, University of Michigan Ann Arbor, MI, USA; ^3^Department of Chemical Engineering, University of Michigan Ann Arbor, MI, USA; ^4^Electrical Engineering and Computer Science, University of Michigan Ann Arbor, MI, USA; ^5^Department of Biomedical Engineering, University of Michigan Ann Arbor, MI, USA; ^6^Department of Materials Science and Engineering, University of Michigan Ann Arbor, MI, USA; ^7^Department of Macromolecular Science and Engineering, University of Michigan Ann Arbor, MI, USA; ^8^Michigan Center for Integrative Research in Critical Care, University of Michigan Ann Arbor, MI, USA; ^9^Michigan Institute of Translational Nanotechnology, University of Michigan Ann Arbor, MI, USA

**Keywords:** Carbon nanotubes, Intravitreal Injection, LHBETATAG Transgenic Mice Retinoblastoma Model, Nanoparticle, Nanotubes, Retinoblastoma

## Abstract

**Purpose:**

To evaluate the penetration of carbon nanotubes (CNTs) throughout retinoblastoma in a transgenic mice model.

**Methods:**

CNTs functionalized with fluorescein isothiocyanate and targeting ligands biotin (CTN-FITC-Bio, 0.5mg/ml), or folic acid (CNT-FITC-FA, 0.5mg/ml) were injected into the vitreous of one eye of LH${}_{\mathrm{BETA}}$T${}_{\mathrm{AG}}$ transgenic mice. Other eye did not receive any injection and was used as control. Three mice were sacrificed at days 1, 2, and 3. Eyes were enucleated and stained with 4,6-diamidino-2-phenylindole. The sections were imaged by fluorescent microscope. The images were transformed into grey-scale in MATLAB for intensity analysis. Background intensity was normalized by marking squares outside the eyeball and using the mean intensity of these squares. Fluorescent intensity (FI) for each image was measured by calculating the intensity of a same-sized square within retinoblastoma.

**Results:**

Nine eyes of nine mice were included in each CNT-FITC-Bio and CNT-FITC-FA groups. The mean FI in CNT-FITC-Bio was 52.08 ± 6.33, 53.62 ± 9.00, and 65.54 ± 5.14 in days 1, 2, and 3, respectively. The mean FI in CNT-FITC-FA was 50.28 ± 7.37, 59.21 ± 6.43, and 58.38 ± 2.32 on days 1, 2, and 3, respectively. FI was significantly higher in eyes injected with CNT-FITC-Bio and CNT-FITC-FA compared to the control eyes (*P* = 0.02). There was no difference in FI between eyes with CNT-FITC-Bio and CNT-FITC-FA, and FI remained stable on days 1–3 in CNT-FITC-Bio, CNT-FITC-FA, and control eyes (*P* > 0.05).

**Conclusion:**

We observed higher FI in eyes with CNT-FITC-Bio and CNT-FITC-FA compared to control eyes, showing penetration of CNTs throughout retinoblastoma. CNTs can be a carrier candidate for imaging or therapeutic purposes in retinoblastoma.

##  INTRODUCTION

Retinoblastoma, a potentially deadly cancer, is the most common intraocular cancer of childhood.^[1]^ Within the last decade, the use of intraarterial and intravitreal chemotherapies significantly changed the management of retinoblastoma. Prior to intraarterial chemotherapy, systemic chemotherapy provided almost 100% globe salvage in group A, B, and C eyes when coupled with laser and cryotherapy and 48% in group D eyes.^[2,3,4]^ The beneficial effect of intraarterial chemotherapy was more pronounced in group D eyes by improving the globe salvage rate up to 100%.^[5,6,7,8]^ However, advanced group D eyes with vitreous seeds and group E eyes continued to be a challenging problem. The use of intravitreal injection especially improved the globe salvage rate in group D eyes with extensive vitreous seeds and group E eyes increasing the globe salvage rate from 27% to 73%.^[9]^ The recurrence of main tumor rather than vitreous seeds was reported to be the reason of failure in globe salvage, suggesting the lack of penetration of chemotherapeutic agents in the main tumor.^[10]^

Carbon nanotubes (CNTs) are unique tubular, hollow nanostructures that have been extensively utilized in biomedical applications including implantable devices.^[11,12]^ They can be single-walled, double-walled, or multi-walled with diameters from < 1 nm up to 100 nm and lengths from 100 nm to microns. *In vivo* and *in vitro* studies showed that CNTs have efficient drug-loading capacity with high length/diameter ratio and multifunctional surface chemistry.^[11,12][13][14]^ In addition, they are biocompatible.^[11,13,14]^ All these features make CNTs an ideal candidate for consideration for drug delivery or tumor imaging. The most of current biodistribution data of CNTs has been obtained based on the systemic administration in animal models.^[15,16]^ By using a three-dimensional (3D) hepatocellular carcinoma tissue culture, Wang et al^[17]^ showed that the surface of CNTs can be engineered to enable deep and fast penetration into tissues. This is achieved by combining the classical 3D diffusion through the interstitial gaps with accelerated two-dimensional (2D) diffusion of the CNTs over the cellular membranes. The combination of deep penetration into the tumor with the ability of CNTs to carry anticancer drugs make them promising candidates for both the diagnostics and treatment of hard-to-reach intraocular tumors.

Retinoblastoma cells have a specialized high affinity carrier-mediated system for folic acid and biotin uptake.^[18,19]^ Therefore, folic acid and biotin can be ideal targets to increase CNTs uptake. In this study, we injected CNTs functionalized with ligands fluorescein isothiocyanate (FITC) and folic acid (CNT-FITC-FA) or biotin (CNT-FITC-Bio) into the vitreous of one eye with LH${}_{\mathrm{BETA}}$T${}_{\mathrm{AG}}$ transgenic retinoblastoma model and compared the fluorescein intensity between the injected and uninjected control eyes to evaluate the ability of CNTs to penetrate through the retinoblastoma tumor when injected intravitreally.

##  METHODS 

### Animals

All experiments are performed in accordance with the Association for Research in Vision and Ophthalmology (ARVO) statement for the Use of Animals in Ophthalmic and Visual Research. The protocol was approved by the University Committee on Use and Care of Animals of the University of Michigan. All surgeries were performed under ketamine and xylazine anesthesia, and all efforts were made to minimize suffering. We used the LH${}_{\mathrm{BETA}}$T${}_{\mathrm{AG}}$ transgenic mice as retinoblastoma animal mode at 8–10 weeks old. Eye tumors in the LH${}_{\mathrm{BETA}}$T${}_{\mathrm{AG}}$ transgenic mouse model showed the histological features of human retinoblastoma with endophytic and exophytic growth with invasion of the retina, choroid, and optic nerve.^[20]^ This animal model has been extensively characterized and develops bilateral multifocal retinal tumors that are stable and grow at the predictable rate.^[20]^ In this model, retinoblastoma develops when the mice is about six weeks old. When the mice reach the age of 8–10 weeks, about half of the globe is filled with the tumor, and at the age of 12–14 weeks, the entire mice globe is filed with retinoblastoma. Examination of each mice was performed and it was confirmed that retinoblastoma tumor fills about 50% of the globe.

#### Preparation of targeted carbon nanotubes functionalized with fluorescein isothiocyanate and biotin (CNT-FITC-Bio), and fluorescein isothiocyanate and folic acid (CNT-FITC-FA)

CNTs were targeted by covalent attachment of biotin and folic acid. Receptors for biotin and folic acid are more highly overexpressed on retinoblastoma cells than retinal pigment epithelium.^[21]^ They were also attached with FITC to image them and to evaluate their penetration. In short, 0.5 mg CNTs with an average diameter of 1.2 nm and a length of 1000 nm (0.5 mg/mL, P3SWNT with 1.0–3.0 atomic % carboxylic acid, Carbon Solutions, Inc.) were dispersed in phosphate-buffered saline (PBS) buffer followed by incubation with 8 mg of 1-ethyl-3-(3-(dimethylamino)propyl) carbodiimide (EDAC) for 1 min at room temperature, after which samples were immediately vortexed. Next, Biotin and FITC (Life Technologies, CA) (2 μg in 20 μL of dimethylformamide) were added together, and the resulting mixture was allowed to react for an additional 2 hours at 37°C in a rotator rocker. These samples were then washed by PBS and centrifuged at 1300 rpm for 20 min for three times to remove unbound antibodies and excess FITC in Centricon YM-50 tubes (MilliporeSigma, MA), and the resulting CNT-FITC-Bio were suspended in 1 mL of serum-free Eagle's Minimum Essential Medium (EMEM) and used immediately. Similar procedure was applied to prepare CNTs functionalized with folic acid to prepare the CNT-FITC-FA.

### Surgical Technique

In this experiment, 1 µl of 0.5 mg/mL targeted CNT-FITC-Bio, and CNT-FITC-FA were injected into the vitreous of one eye of LH${}_{\mathrm{BETA}}$T${}_{\mathrm{AG}}$ transgenic mice. The other eye was not injected and was used as control. In each group of CNT-FITC-Bio (nine eyes) and CNT-FITC-FA (nine eyes), nine eyes of nine mice were used. The control group comprised the uninjected eyes of nine mice. Vitreous injections were performed by an experienced team member (CL) under direct visualization with the operating microscope, after dilating the pupil and confirming that injection was into the vitreous cavity, but not into the tumor. During the procedure, the tip of the needle was constantly monitored. Three mice were sacrificed at each day 1, 2, and 3, and eyes were enucleated.

### Histopathological Preparation 

Mice eyes were fixated with 10% formaldehyde in phosphate-buffered saline (PBS) for histology or with 4% paraformaldehyde in PBS followed by incubating with 30% sucrose in PBS. Eyes were embedded in optimal cutting temperature (OCT) compound and cryosectioned. They were stained with 4,6-diamidino-2-phenylindole (DAPI, 1 mg/mL in PBS; Sigma-Aldrich) to visualize cell nuclei.

### Image Analysis

**Figure 1:**
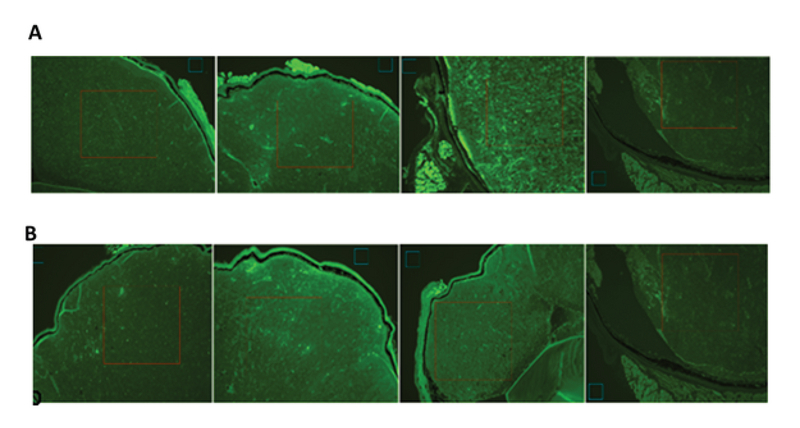
(A) From left to right, eyes injected with CNT-FITC-Bio on days 1, 2, and 3 and control eye (×10, DAPI). (B) From left to right eyes injected with CTN-FITC-FA on days 1, 2, and 3 and control eye (×10, DAPI). The blue squares in each image denotes the area in glass region, which is used for normalization. The red squares were used to calculate the averaged intensity within tumor. FITC-functionalized CNTs appear as bright spots throughout the retinoblastoma tumor in colored images.

Each globe was sectioned in to 5 µm thick sections. Five sections from each globe were evaluated and count of these five sections were averaged. The stained sections were imaged by Olympus BX-51 fluorescent microscope under 10× magnification. The fluorescent CNTs were excited by 480 nm light with the same laser power and the camera exposure was kept the same all the time (which is 102.6 ms). The images were transformed into grey-scale images in MATLAB for intensity analysis. In order to compare the intensity, all the images were normalized. For normalization, a blue square region (blue square marked in Figure 1) outside the eyeball area was marked in each eye, and the mean intensity within these same-sized blue squares were calculated for each eye. It is understandable that the glass region should reflect same, fixed light intensity. Therefore, all the images were normalized by setting the mean intensity of these blue squares to be the same. Finally, the fluorescent intensity (FI) for each image was calculated by calculating the intensity of a same-sized red square region (red square marked in Figure 1) within the eyeball area. We avoided the muscle cell area which could also show strong fluorescents. It is worth noting that the size of this square was determined by the largest possible area among the images. In order to compensate the false-positive results resulting from the photoconversion of DAPI due to blue excitation and green emission as reported by Jez et al,^[22]^ we analyzed both control and injected eyes, and compared them with each other.

**Figure 2:**
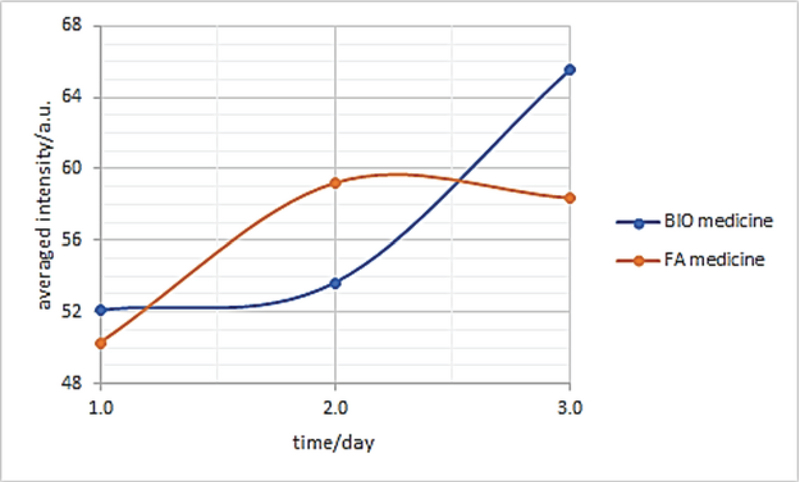
The change of average intensity of CNTs functionalized with fluorescein isothiocyanate and biotin, and fluorescein isothiocyanate and folic acid over the time.

### Statistical Analysis

The Difference in the fluorescein intensity between eyes injected with CNT-FITC-Bio and CNT-FITC-FA, and the control eyes were compared by using the Mann–Whitney U test. Similarly, the difference in the fluorescein intensity between eyes injected with CNT-FITC-FA and CNT-FITC-Bio were also compared by using Mann-Whitney U test. The change in the fluorescein intensity in eyes injected with CNT-FITC-FA and CNT-FITC-Bio and control eyes at different days were evaluated by using repeated measures ANOVA test.

##  RESULTS

Nine eyes of nine mice were included in each group of CNT-FITC-Bio and CNT-FITC-FA. In all eyes of LH${}_{\mathrm{BETA}}$T${}_{\mathrm{AG}}$ transgenic mice, retinoblastoma tumor occupied about 50% of mice eye. We found that the fluorescence intensity was higher in retinoblastoma tumor in eyes injected with CNT-FITC-FA and CNT-FITC-Bio than the uninjected control eyes (Table 1). The FI in the retinoblastoma tumor remained about the same on days 1 and 2, and mildly increased on day 3 for CNT-FITC-Bio (Figure 1A). The FI increased on day 2 and remained about the same on day 3 for CNT-FITC-FA (Figure 2). We also observed that both CNT-FITC-Bio and CNT-FITC-FA passed through the retinoblastoma and stained the retinal pigment epithelium, showing their penetration through the tumor (Figure 1B). The mean fluorescein intensity was significantly higher in eyes injected with CNT-FITC-Bio and CNT-FITC-FA compared to the uninjected control eyes (*P* = 0.02). We did not observe any difference in the mean fluorescein intensity between CNT-FITC-Bio and CNT-FITC-FA groups (*P* > 0.05). There was no significant change in the fluorescein intensity at different days in eyes injected with CNT-FITC-Bio and CNT-FITC-FA and the uninjected control eyes (*P* > 0.05).

**Table 1:** Averaged intensities measured in eyes injected with CNT-FITC-Bio and CNT-FITC-FA


	**Day1**	**Day2**	**Day3**	**Control**
Averaged intensity CNT-FITC-Bio sample/a.u.	52.08 ± 6.33 (52,42.60–56.55)	53.62 ± 9.00 (53, 47.25–60)	65.54 ± 5.14 (62, 58.26–84.25)	34.47 ± 6.67 (34, 29.76–39.19)
Averaged intensity CNT-FITC-FA sample/a.u.	50.28 ± 7.37 (50, 45.07-55.49)	59.21 ± 6.43 (55, 50.11–73.19)	58.38 ± 2.32 (58, 56.74–60.01)	34.47 ± 6.67 (34, 29.76–39.19)
*The fluorescein intensity was significantly higher in eyes injected with CNT-FITC-Bio and CNT-FITC-FA compared to the control uninjected eyes (*p* = 0.02). **There was no difference in fluorescein intensity between CNT-FITC-Bio and CNT-FITC-FA groups (*p* > 0.05). ***There was no significant change in fluorescein intensities at different days in the eyes with CNT-FITC-Bio and CNT-FITC-FA and the uninjected control eyes (*p* > 0.05).				

Beside the tumor, we did not observe staining in the lens, iris, or cornea. There was no dose- or procedure-related complications (cataract or retinal detachment).

##  DISCUSSION 

Carbon nanotubes have gained tremendous attention as drug carriers or imaging tools due to their unique characteristics such as high surface area, enhanced cellular uptake, and the possibility to be easily conjugated with many therapeutic or diagnostic agents.^[15,16][17]^ Previous studies about CNTs were usually based on animal studies or 3D tissue cultures.^[15,16][17]^ In these studies, CNTs were delivered systemically to the tumor through the blood vessels and penetrated through the vessel walls or added to the 3D tissue culture medium. In this study, intravitreal CNTs penetrated and passed through the retinoblastoma tumor and reached the retinal pigment epithelium layer. This was the first study that showed that CNTs can penetrate and diffuse through a solid tumor such as retinoblastoma when injected intravitreally. Wang et al^[17]^ investigated the diffusional transport of CNTs in 3D tissue replica of hepatocellular carcinoma and found that diffusion coefficients of CNTs were high and comparable to the diffusion rates of similarly charged molecules with molecular weights 10000x lower. The diffusivity of CNTs in tissues was enhanced after functionalization with transforming growth factor β1. The high diffusion coefficient was attributed to the planar diffusion of CNTs along cellular membranes reducing effective dimensionality of diffusional space and electrostatic repulsion between CNT and cellular membrane. Although the penetration of CNTs remained stable on the first and second days in the eyes injected with CNT-FITC-Bio with mild increase on day 3, and the penetration increased on day 2 and remained stable on day 3 in eyes injected with CNT-FITC-FA, these changes were not found to be statistically significant. Studies with larger number of animals and longer follow-up are needed to confirm these findings and if CNTs will remain stable inside the retinoblastoma over time.

With the introduction of well-tolerated intravitreal injection technique in retinoblastoma, intravitreal chemotherapy gained an attention by improving the control of vitreous seeds which were the main reason of treatment failures.^[23,24,25]^ Abramson et al^[26]^ reviewed eyes treated with intravitreal chemotherapy for indications other than vitreous seeds including subretinal seeds and recurrent retinal tumors in 56 eyes of 52 patients and found the recurrence rate of retinal tumors in 19% of eyes and subretinal seeds in 11% of eyes. They concluded that intravitreal chemotherapy could be considered as adjuvant therapy in globe-sparing treatment. The penetration and diffusion of CNTs in retinoblastoma in animal models suggest that they could be an ideal carrier for chemotherapeutic agents in retinoblastoma. This might provide treatment of vitreous or subretinal seeds or retinoblastoma.

There are some limitations of our study. This was a pilot study that was performed in small numbers of animals and these eyes were evaluated on days 1, 2, and 3. We did not do any electron microscopy to show the distribution of CNTs. However, higher fluorescein intensity throughout retinoblastoma tumors showed that CNTs have significantly higher penetration throughout the retinoblastoma tumors. In this study, we did not evaluate the distribution of CNTs on the other ocular structures in detail as well as their toxicities. Future studies will be needed to address these issues.

In conclusion, we showed that CNTs can penetrate through the retinoblastoma tumor in a transgenic retinoblastoma model when administrated into the vitreous. There was no difference in tumor penetration between the CNT-FITC-Bio and CNT-FITC-FA groups. The deep penetration into the tumor with the ability of CNTs to carry chemotherapeutic agents make them promising carrier candidates for both diagnostics and treatment of these hard-to-reach intraocular tumors.

##  Financial Support and Sponsorship 

This study was supported by Richard N and Marilyn K Witham Professorship.

##  Conflicts of Interest

There are no conflicts of interest.

## References

[1] Young JL, Smith MA, Roffers SD, Liff JM, Bunin GR. Retinoblastoma. In: Ries LA, Smith MA, Gurney JG, Linet M, Tamra T, Young JL, et al., editors. Cancer incidence and survival among children and adolescents: United States SEER program 1975–1995. Maryland: National Cancer Institute, SEER Program; 2012;73-79.

[2] Shields CL, DePotter P, Himelstein BP, Shields JA, Meadows AT, Maris JM. Chemoreduction in the initial management of intraocular retinoblastoma. *Arch Ophthalmol* 1996;114:1330–1338. 10.1001/archopht.1996.01100140530002 8906023

[3] Murphree AL, Villablanca JG, Deegan WF, Sato JK, Malogolowkin M, Fisher A, et al. Chemotherapy plus local treatment in the management of intraocular retinoblastoma. *Arch* *Opthalmol* 1996;114:1348–1356. 10.1001/archopht.1996.01100140548005 8906025

[4] Gallie BL, Budning A, DeBoer GKoren G, Verje, Thiessen JJ, e Z, et al. Chemotherapy with focal therapy can cure intraocular retinoblastoma without radiotherapy. *Arch Ophthalmol* 1996;114:1321–1328. 10.1001/archopht.1996.01100140521001 8906022

[5] Shields CL, Jorge R, Say EA, Magrath G, Alset A, Caywood E, et al. Unilateral retinoblastoma managed with intravenous chemotherapy versus intra-arterial chemotherapy. Outcomes based on the international classification of retinoblastoma. *Asia Pac J Ophthalmol* 2016;5:97–103. 10.1097/APO.0000000000000172 26765038

[6] Manjandavida FP, Stathopoulos C, Zhang J, Honavar SG, Shields CL. Intra-arterial chemotherapy in retinoblastoma - a paradigm change. *Indian J Ophthalmol* 2019;67:740–754. 10.4103/ijo.IJO_866_19 PMC6552585 31124482

[7] Munier FL, Mosimann P, Puccinelli F, Gaillard MC, Stathopoulos C, Houghton S, et al. First-line intra-arterial versus intravenous chemotherapy in unilateral sporadic group D retinoblastoma: evidence of better visual outcomes, ocular survival and shorter time to success with intra-arterial delivery from retrospective review of 20 years of treatment. *Br J Ophthalmol* 2017;101:1086–1093. 10.1136/bjophthalmol-2016-309298 PMC5537510 27927678

[8] Abramson DH, Marr BP, Dunkel IJ, Brodie S, Zabor EC, Driscoll SJ, et al. Intra-arterial chemotherapy for retinoblastoma in eyes with vitreous and/or subretinal seeding: 2-year results. Intra-arterial chemotherapy for retinoblastoma in eyes with vitreous and/or subretinal seeding: 2-year result. *Br J Ophthalmol *2012;96:499–502. 10.1136/bjophthalmol-2011-300498 22053101

[9] Shields CL, Alset AE, Say EA, Caywood E, Jabbour P, Shields JA*. *Retinoblastoma control with primary intra-arterial chemotherapy: outcomes before and during the intravitreal chemotherapy era. *J Pediatr Ophthalmol Strabismus *2016;53:275–284. 10.3928/01913913-20160719-04 27486728

[10] Ghassemi F, Amoli FA. Pathological findings in enucleated eyes after intravitreal melphalan injection. *Int Ophthalmol* 2014;34:533–540. 10.1007/s10792-013-9851-2 24043335

[11] REF Tang Z, Wang Y, Podsiadlo P, Kotov NA. Biomedical applications of layer-by-layer assembly: from biomimetics to tissue engineering. *Adv Mater* 2006;18:3203–3224.

[12] Gheith MK, Pappas TC, Liopo AV, Sinani V, Shim BS, Motamedi M, et al. Stimulation of neural cells by lateral currents in conductive LBL films of single-walled carbon nanotubes. *Adv Mater* 2006;18:2975–2979.

[13] Mohajeri M, Behnam B, Sahebkar A. Biomedical applications of carbon nanomaterials: drug and gene delivery potentials. *J Cell Physiol* 2018;234:298–319. 10.1002/jcp.26899 30078182

[14] Yang K, Liu Z. In vivo biodistribution, pharmacokinetics, and toxicology of carbon nanotubes. *Curr Drug Metabol *2012;13:1057–1067. 10.2174/138920012802850029 22380009

[15] Singh R, Pantarotto D, Lacerda L, Patorin G, Klumpp C, Prato M, et al. Tissue Biodistribution and Blood Clearance Rates of Intravenously Administered Carbon Nanotube Radiotracers. *Proc Natl Acad Sci USA* 2006;103:3357–3362. 10.1073/pnas.0509009103 PMC1413890 16492781

[16] Sager TM, Wolfarth MW, Andrew M, Hubbs A, Friend S, Chen TH, et al. Effect of Multi-Walled Carbon Nanotube Surface Modification on Bioactivity in the C57bl/6 Mouse Model. *Nanotoxicology* 2014;8:317–327. 10.3109/17435390.2013.779757 PMC4669410 23432020

[17] Wang Y, Bahng JH, Che Q, Han J, Kotov NA. Anomalously fast diffusion of targeted carbon nanotubes in cellular spheroids. *ACS Nano* 2015;9:8231–8238. 10.1021/acsnano.5b02595 PMC11135955 26181892

[18] Kansara V, Paturi D, Luo S, Gaudana R, Mitra AK. Folic acid transport via high affinity carrier-mediated system in human retinoblastoma cells. *Int J Pharm* 2008;355:210–219. 10.1016/j.ijpharm.2007.12.008 PMC4516230 18207340

[19] Kansara V, Luo S, Balasubrahmanyam B, Pal D, Mitra AK. Biotin uptake and cellular translocation in human derived retinoblastoma cell line (Y-79): a role of hSMVT system. *Int J Pharm* 2006;312:43–52. 10.1016/j.ijpharm.2005.12.045 16459033

[20] Albert DM, Griep AE, Lambert PF, Howes KA, Windle JJ, Lasudry JG. Transgenic models of retinoblastoma; what they tell us about its cause and treatment. *Trans Am Ophthalmol Soc* 1994;92:385–400. PMC1298518 7886874

[21] Jwala J, Vadlapatla RK, Vadlapudi AD, Boddu SH, Pal D, Mitra AK. Differential expression of folate receptor-alpha, sodium-dependent multivitamin transporter, and amino acid transporter (B (0, +)) in human retinoblastoma (Y-79) and retinal pigment epithelial (ARPE-19) cell lines. *J Ocul Pharmacol Ther* 2012;28:237–244. 10.1089/jop.2011.0155 PMC3361182 22304562

[22] Jez M, Bas T, Veber M, Košir A, Dominko T, Page R, et al. The hazards of DAPI photoconversion: effects of dye, mounting media and fixative, and how to minimize the problem. *Histochem Cell Biol* 2013;139:195–204. 10.1007/s00418-012-1039-8 23064788

[23] Francis JH, Roosipu N, Levin AM, Brodie SE, Dunkel IJ, Gobin YP, et al. Current treatment of bilateral retinoblastoma: the impact of intraarterial and intravitreous chemotherapy. *Neoplasia* 2018;20:757–763. 10.1016/j.neo.2018.05.007 PMC6020084 29940303

[24] Munier FL, Gaillard MC, Balmer A, Soliman S, Podilsky G, Moulin AP, et al. Intravitreal chemotherapy for vitreous disease in retinoblastoma revisited: from prohibition to conditional indications. *Br J Ophthalmol* 2012;96:1078–1083. 10.1136/bjophthalmol-2011-301450 22694968

[25] Shields CL, Lally SE, Leahey AM, Jabbour PM, Caywood EH, Schwendeman R, et al. Targeted retinoblastoma management: when to use intravenous, intra-arterial, periocular, and intravitreal chemotherapy. *Curr Opin Ophthalmol* 2014;25:374–385. 10.1097/ICU.0000000000000091 25014750

[26] Abramson DH, Ji X, Francis JH, Catalanotti F, Brodie SE, Habib L. Intravitreal chemotherapy in retinoblastoma: expended use beyond intravitreal seeds. *Br J Ophthalmol* 2018; June 6 [Epub ahead of print]. 10.1136/bjophthalmol-2018-312037 PMC8132339 29875233

